# 3D printed infliximab suppositories for rectal biologic delivery

**DOI:** 10.1016/j.ijpx.2023.100176

**Published:** 2023-03-08

**Authors:** Atheer Awad, Alvaro Goyanes, Mine Orlu, Simon Gaisford, Abdul W. Basit

**Affiliations:** a Department of Pharmaceutics, UCL School of Pharmacy, University College London, 29-39 Brunswick Square, London, WC1N 1AX, UK; b FabRx Ltd., Henwood House, Henwood, Ashford, Kent, TN24 8DH, UK; c Departamento de Farmacología, Farmacia y Tecnología Farmacéutica, I+D Farma (GI-1645), Facultad de Farmacia, Materials institute (iMATUS) and Health Research Institute (IDIS), Universidade de Santiago de Compostela, 15782 Santiago de Compostela, Spain

**Keywords:** Ulcerative colitis (UC) and Crohn's disease (CD), Rectal drug delivery, Biologicals and monoclonal antibodies, Pressure-assisted syringe, Digital medicine production, Tumour necrosis factor-alpha (TNF-alpha) inhibitors, Extrusion-assisted additve manufacturing of a suppository formulation

## Abstract

Infliximab is a monoclonal antibody that plays an important role in the management and treatment of chronic inflammatory bowel diseases (IBD). Due to its macromolecular structure, its delivery through the oral route is challenging, limiting its administration to only via the parenteral route. The rectal route offers an alternative way for administering infliximab, allowing it to be localised at the disease site and circumventing its passage across the alimentary canal and thus, maintaining its integrity and bioactivity. Three-dimensional (3D) printing is an advanced production technology that permits the creation of dose-flexible drug products from digital designs. The current study assessed the feasibility of utilising semi-solid extrusion 3D printing for the fabrication of infliximab-loaded suppositories for the local treatment of IBD. Various printing inks composed of Gelucire® (48/16 or 44/14) mixed with coconut oil and/or purified water were investigated. It was shown that following reconstitution in water, the infliximab solution can be directly incorporated into the printing ink of Gelucire® 48/16 and can withstand the extrusion process, resulting in well-defined suppositories. Since water content and temperature are critical for safeguarding infliximab's potency, the effect of changing the composition of the printing inks and printing parameters on infliximab's biologic efficiency was evaluated by measuring its binding capacity (i.e., the amount of infliximab that actively binds to its antigen to exert an effect). Despite drug loading assays showing that infliximab remains intact following printing, it was found that the incorporation of water in isolation results in only ∼65% binding capacity. However, when oil is added to the mixture, infliximab's binding capacity increases up to ∼85%. These promising results demonstrate that 3D printing has the potential to be exploited as a novel platform for fabricating dosage forms containing biopharmaceuticals, avoiding patients' compliance issues observed with injectables and addressing their unmet needs.

## Graphical Abstract

**Figure:**
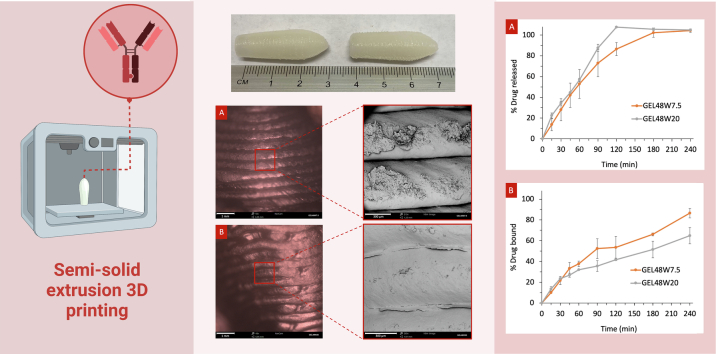

## 1 Introduction

Inflammatory bowel disease (IBD) refers to a group of chronic inflammatory conditions that affect the intestinal mucosa of the gastrointestinal (GI) tract (Awad et al., 2022; Cosnes et al., 2011). It is differentiated into two main conditions, ulcerative colitis (UC) and Crohn's disease (CD), with each having its unique clinical manifestations (Collnot et al., 2012). First-line treatments of IBD include mesalazine and corticosteroids (McCoubrey et al., 2023; Okobi et al., 2021). Despite their proven efficacy and fast remission rates, mesalazine and corticosteroids do not show a therapeutic response in 30–60% and 20–40% of UC patients, respectively (Endo et al., 2016; Faubion Jr et al., 2001; Ho et al., 2006). This requires the administration of alternative treatments, such as tumour necrosis factor alpha (TNF-α) monoclonal antibodies (e.g., infliximab) or immunosuppressants (e.g., cyclosporin). Although both medications have shown equivalent effectiveness at the treatment of acute severe ulcerative colitis (ASUC), infliximab offers a less complex treatment regimen, making its use preferable over that of cyclosporin (Lamb et al., 2019).

Infliximab is a chimeric IgG1 monoclonal antibody (mAB) that reduces serum levels of pro-inflammatory cytokines (e.g., interleukin-6 (IL-6), interferon-gamma (IFN-γ)) and chemokines (e.g., monocyte chemoattractant protein-1 (MCP-1), macrophage inhibitory protein-2 (MIP-2)) (Markham and Lamb, 2000). Whilst its macromolecular structure renders it highly potent and specific, it also impedes its permeation through biological barriers and makes it more prone to degradation in the GI tract. Due to that, infliximab is typically administered intravenously (IV) or subcutaneously (SC), however, this has been associated with high incidence of systemic adverse events (Yadav et al., 2016). Moreover, although, unlike the IV route, SC formulations can be self-administered without the need for professional personnel, both routes of administration are considered invasive (Shepherd and Shepherd, 2020), affecting patients' compliance to treatments (Antosova et al., 2009; Brod et al., 2008; Wentworth et al., 2018). With the challenges posed by the oral, IV and SC routes, it may be advantageous to explore a different route for the administration of infliximab. In this regard, the rectal route offers a suitable alternative, permitting the localisation of the drug at the disease site, whilst avoiding passage through the alimentary canal and thus, potentially maintaining the biologic drug's integrity (Hua, 2019). In fact, the rectum has several features that makes it well suited for the administration of biopharmaceuticals. This includes being rich in blood vessels, the absence of GI juices and enzymes, and abundance of mucosal tissues with T lymphocytes. Furthermore, the pH in the rectum has been reported to be between 6.7 and 7.4 (Fallingborg, 1999; Yan et al., 2015), making it appropriate for maintaining the chemical stability of infliximab and preventing its denaturation (Yadav et al., 2016). Furthermore, rectal formulations are less invasive than injections and infusions, and can be self-administered by patients, making them more appealing to some patients over injectable therapies (Chu and Traverso, 2022; Mahajan et al., 2017).

Therefore, herein we created suppositories containing infliximab for the treatment of IBD. Currently, suppositories are manufactured through the fusion moulding method; however, this generally requires the use of moulds that have limited lifespan and are dose inflexible. Three-dimensional (3D) printing is a relatively new manufacturing tool being investigated in the pharmaceutical sector for the preparation of customised dosages (Awad et al., 2023a; Cui et al., 2021; Lafeber et al., 2022; Xu et al., 2021). Semi-solid extrusion (SSE) is a material extrusion 3D printing technology (Awad et al., 2018) that can shape semi-solid materials (e.g., pastes, gels, and wax) into 3D objects by extruding them through a heated orifice (Domsta et al., 2022; Seoane-Viaño et al., 2021b; Sjöholm et al., 2022; Zhu et al., 2022). Unlike analogous 3D printing technologies that necessitate the use of elevated temperatures, SSE 3D printing utilises low temperatures, making it well suited for use with thermosensitive active pharmaceutical ingredients (APIs) (Evans et al., 2021; Rodríguez-Pombo et al., 2022). Building on our previous work where we have shown that SSE 3D printed suppositories can be used for delivering small drug molecules (e.g., tacrolimus (Seoane-Viaño et al., 2020; Seoane-Viaño et al., 2021a), tofacitinib citrate and budesonide (Awad et al., 2023b)), herein we demonstrate that this flexible platform can be expanded for the delivery of biopharmaceuticals which are more challenging to produce.

Thus, the aim of this study is to use SSE 3D printing for the fabrication of infliximab-loaded suppositories with varying water and coconut oil content, wherein their effect on printing, disintegration and drug release was examined. Infliximab's stability was assessed using size exclusion chromatography (i.e., chromatographic analytic technique that separates molecules in solution based on their size) and its biologic activity was measured by calculating its binding capacity (i.e., the amount of infliximab that actively binds to an antigen to exert an effect) using enzyme-linked immunosorbent assay (ELISA). The emulsions formed by the suppositories were also investigated for their self-emulsification time, globule sizes and stability.

## 2 Materials and methods

### 2.1 Materials

Infliximab (European Pharmacopeia reference standard) and coconut oil (from *Cocos nucifera*) were purchased from Merck Life Sciences, UK. Gelucire® 48/16 (also known as Polyethylene glycol-32 (PEG-32) stearate; and is composed of PEG-32 (molecular weight (MW) 1500 g/mol) esters of palmitic (C_16_) and stearic (C_18_) acids) and 44/14 (also known as lauroyl PEG-32 glycerides; and is composed of PEG-32 (MW 1500 g/mol) mono- and diesters of lauric acid (C_12_) mixed with a small portion of mono, di- and triglycerides) were kindly donated by Gattefossé, France. The recombinant human TNF-α protein was acquired from R&D Systems, Bio-Techne, UK. The goat anti-human IgG Fc secondary antibody Horseradish Peroxidase (HRP) conjugate was purchased from ThermoFisher Scientific, UK. Bovine Serum Albumin (BSA), Tween 20 and Dulbecco's Phosphate Buffered Saline (PBS) were obtained from Sigma-Aldrich, UK. The enhanced K-BLUE 3,3',5,5' tetramethylbenzidine (TMB) substrate and RED STOP solutions were bought from Neogen Europe Limited, Scotland. Ultrapure water was acquired by filtering tap water through a PURELAB Chorus 1 complete water purification system (Veolia Water Technologies, UK).

### 2.2 Semi-solid extrusion (SSE) 3D printing

To prepare the formulations, Gelucire® and coconut oil were melted at 60 °C and mixed in a glass beaker at a speed of 400 rpm using a Super-Nuova Multi-Position Digital Stirring Hotplate (Thermo Fisher Scientific, Massachusetts, USA) (Table 1). Once all the components were mixed, infliximab and any additional ultrapure water were stirred in for 5 min. The mixtures were immediately transferred to a 20 mL Injekt® disposable syringe (Braun, Germany) and allowed to solidify at room temperature then stored at −20 °C.

**Table 1:** Summary of the compositions of the formulations used for printing.

Formulation	Gelucire® 48/16 (%*w*/w)	Gelucire® 44/14 (%w/w)	Coconut oil (%w/w)	Purified water (%w/w)	Infliximab (%w/w)	Preparation temperature (°C)	Printing temperature (°C)
Gel44W0	0	79.80	19.96	0	0.24⁎	60	– ^
Gel44W7.5	0	79.80	12.48	7.48§	0.24‡	60	–
Gel44W20	0	79.80	0	19.96§	0.24‡	60	–
Gel48W0	79.80	0	19.96	0	0.24⁎	60	– ^
Gel48W7.5	79.80	0	12.48	7.48§	0.24‡	60	38
Gel48W20	79.80	0	0	19.96§	0.24‡	60	30

⁎ *Unreconstituted, lyophilised powder was used.*

‡ *Reconstituted infliximab solution was used.*

§ *This weight includes the weight of purified water in the reconstituted solution containing the required weight of infliximab.*

^ *Infliximab powder did not dissolve in the lipid mixture; thus, formulation printing was not attempted.*

For the reconstituted infliximab solution, the manufacturer's instructions were followed. Each infliximab vial was reconstituted with 2.5 mL of purified water to produce a concentrated solution with a mAB concentration of ∼35.6 mg/mL infliximab. The infliximab dose (5 mg) was selected according to previously published literature (Maurer et al., 2016).

The pre-filled syringes were fitted with a tapered 0.024″ - 0.61 mm extrusion tip (Fisnar, UK) and placed into the SSE printhead of the M3DIMAKER 2 3D printer (FabRx Ltd., UK). The previously prepared .gcode of the vertical suppositories' 3D models (Dimensions: 12 mm × 36 mm (Seoane-Viaño et al., 2021a)) was loaded into the printer's software and printed using the following printing parameters: 0.5 mm layer height; 2.4 mm shell thickness; 25 mm/s flow speed; 1.2 mm nozzle size; room temperature of build plate and chamber; printing temperatures as shown in Table 1**.** Finally, the 3D printed suppositories were allowed to solidify at room temperature for ∼2 min and subsequently stored in the freezer at −20 °C to preserve infliximab's integrity.

### 2.3 Characterisation of the 3D printed suppositories

#### 2.3.1 Scanning electron microscopy (SEM)

The suppositories were cut in half, attached to a 25 mm conductive carbon adhesive disc mounted (Taab, UK) on a 25.4 mm short pin aluminium stub (Taab, UK) and coated with 25 nm of gold using a sputter coater. The stub was then placed into a Phenom Pro Desktop Scanning Electron Microscope (Thermo Fisher Scientific, UK) at 10 kV accelerating voltage using secondary electron detection to obtain images of the surface of the suppositories. The optical navigation camera was also used to capture images of the suppositories. ImageJ (Version 1.53 t, Wayne Rasband and contributors National Institutes of Health, Maryland, USA) was used for analysing the layers' height. The mean from10 different measurements was used for each formulation.

#### 2.3.2 Transmission electron microscopy (TEM)

A portion of each suppository (50 mg) was placed in 100 mL of ultrapure water under magnetic stirring at 100 rpm until the emulsion was formed. A drop of diluted SEDDS was then deposited on the carbon-formvar film grid, stained by 1% aqueous solution of phosphotungstic acid and observed after drying. TEM images of emulsion droplets were taken using a CM120 Philips Biotwin transmission electron microscope (Eindhoven, The Netherlands).

#### 2.3.3 Characterisation of emulsions

A portion of each suppository (50 mg) was dissolved in 100 mL of ultrapure water under constant stirring at 100 rpm until the emulsions were formed. Droplet size distribution (i.e., average diameter of the lipid droplets), polydispersity index (PDI; i.e., range of molecular weight distribution of the droplets) and Zeta (ζ) potential (i.e., the charge at the interface of the droplets) were determined using a Zetasizer Nano (Malvern Instrument Limited, UK). All measurements were performed in triplicate.

#### 2.3.4 Fourier transform-infrared spectroscopy (FTIR)

The FTIR spectra were collected using a Spectrum 100 FTIR spectrometer (PerkinElmer, Massachusetts, USA). Lyophilised infliximab powder, reconstituted infliximab solution, Gelucire® 48/16, coconut oil and ultrapure water were measured as the references for the 3D printed formulations. Samples were scanned over a range of 4000–650 cm^−1^ at a resolution of 4 cm^−1^ for 24 scans.

#### 2.3.5 Determination of disintegration time

*In vitro* disintegration testing was performed in distilled water at 37 ± 0.5 °C using a United States Pharmacopeia (USP) disintegration apparatus (ZT43, Copley, UK) with a basket rack assembly and perforated discs, slightly modified to confer with the requirements of the European Pharmacopeia (EP) (European Pharmacopeia, 2022; Seoane-Viaño et al., 2021a). The volume of water in the vessel was adjusted according to USP standards (i.e., when the basket is at the highest position, the wire mesh was at least 15 mm under the liquid surface, and when moved down, it was lowered by 25 mm or more from the beaker's bottom). Three suppositories from each formulation were tested, wherein a perforated disc was placed on top each suppository. Disintegration was considered achieved when the suppository components were separated or when the suppository softened and underwent a considerable change in shape.

#### 2.3.6 Determination of self-emulsification time

Emulsification time of the SEDDS suppositories was determined using a USP II dissolution apparatus. Each formulation was melted and 20 μL of the molten mass was added dropwise to 500 mL of ultrapure water at 37 ± 0.5 °C, gently agitated by rotating the paddles at 50 rpm. Self-emulsification time was recorded as the time taken by each formulation (*n* *=* *3*) to form a clear solution in water.

#### 2.3.7 Drug loading and binding capacity

Drug-loaded suppositories were dissolved in 100 mL of 0.1 M phosphate buffer (pH 8.0) at 37 ± 0.5 °C under continuous magnetic stirring at 100 rpm for 6 h. Size exclusion chromatography (SEC) studies were performed to quantify the amount of infliximab present in the suppositories (*n* *=* *3*) following printing. A HPLC system (Hewlett Packard 1260II Series HPLC system, Agilent Technologies, Cheadle, UK) was used for these studies. The stationary phase consisted of a YMC pack Diol 200 Å column (300 mm × 8.0 mm, 5 μm, 20 nm), wherein 10 μL of each sample was injected. A mobile phase consisting of 50 mM phosphate buffer, 200 mM NaCl (pH 7.0) was injected into the column at a 0.5 mL/min flow rate. The column's temperature was kept at 25 ± 0.5 °C and eluents were screened using a variable wavelength detector at 214 nm.

The binding capacity of infliximab was evaluated using an enzyme-linked immunosorbent assay (ELISA) test carried out in 96-well Nunc MaxiSorp Flat-Bottom plates (Invitrogen, Massachusetts, USA), wherein each well was coated with 100 μL of 1 μg/mL recombinant Human TNF-α Protein antigen diluted in PBS and incubated at 4 °C overnight. Before assay, the contents of the plates were discarded, and the plates were then washed 3 times with 200 μL/well using the washing buffer (0.1% *v*/v Tween 20 in PBS). Subsequently, 200 μL/well of blocking buffer was added and the plates were then incubated at 37 °C for 1 h. Following incubation, the blocking buffer was discarded and 100 μL/well of each sample were added and the plates were incubated at 37 °C for 1 h. Following the incubation, the plates were washed 3 times with 200 μL/well of washing buffer. 100 μL/well of the secondary antibody (Goat Anti-human IgG Fc Secondary Antibody, HRP-conjugated; diluted 20,000 times in assay buffer (PBS + 0.2% *w*/*v* BSA + 0.1% Tween 20)) was then added and the plates were incubated for 45 min at 37 °C. Subsequently, the contents of the wells were discarded and washed 3 times with 200 μL/well of washing buffer. 150 μL/well of K-BLUE TMB substrate was added and the plates were incubated for 10 min at room temperature in the dark. This was followed by stopping the substrate reaction after 10 min through the addition of 50 μL/well of RED STOP solution. Reading was carried out at 650 nm using a Multiskan FC microplate photometer (ThermoScientific, UK), wherein the plate was shaken for 10 s at a slow speed. Infliximab concentrations were extrapolated from a sigmoid curve fit, where data was plotted using GraphPad Prism (Version 9.4.1, GraphPad Software LLC, California, USA).

#### 2.3.8 In vitro drug release

*In vitro* drug release profiles of the 3D printed suppositories were obtained using a USP-II mini paddle apparatus (Model PTWS, Pharmatest, Hainburg, Germany). The studies were conducted in 100 mL 0.1 M phosphate buffer (pH 8.0) and the paddle speed was set at 100 rpm with a temperature of 37 ± 0.5 °C (*n* = 3). At pre-determined time points (15, 30, 45, 90, 120, 180, 240 min), 1 mL aliquots were withdrawn, and samples were analysed using SEC and ELISA as described in Section 2.3.7.

### 2.4 Statistical analysis

One-way analysis of variance (ANOVA) test was used to evaluate the statistical significance of the difference between the obtained results from different formulations (drug loading, binding capacity, disintegration, dissolution, self-emulsification time, droplet size, PDI and ζ potential studies). All analyses were executed using Origin (OriginPro 2019, OriginLab corporation, USA), wherein *P* < 0.05 was regarded as statistically significant.

## 3 Results and discussion

**Fig. 1:**
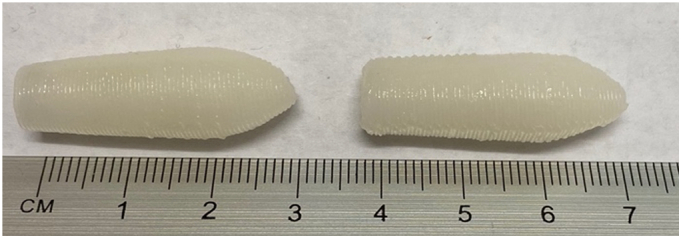
Images of the infliximab-loaded suppositories fabricated using the (left) GEL48W7.5 and (right) GEL48W20 formulations, respectively. Scale shown in cm.

In this study, 3D printed rectal dosage forms loaded with a biopharmaceutical were successfully created using the SSE 3D printing technology (Fig. 1). To maintain the stability of mABs, they are often prepared and stored as lyophilised powders (Wang, 2000). Attempts to dissolve the lyophilised infliximab powder in the suppository base mixture of Gelucire® and coconut oil were unsuccessful (Gel44W0 and Gel48W0). To overcome this, successive formulations were formulated using the reconstituted infliximab solution. Initially, Gelucire® 44/14 and 48/16 were investigated. In particular, the effectiveness of using purified water as a plasticiser alone or in combination with coconut oil was explored. It was found that Gelucire® 44/14 softens to a high extent when mixed with water, resulting in a fluid mixture that is unsuitable for printing (Gel44W7.5 and Gel44W20). This may be attributed to the facts that Gelucire® 44/14 is a water dispersible surfactant and has an onset melting temperature of 38 °C (PharmaExcipients, 2020a). And therefore, when dispersed with water, it starts to soften at or below room temperature. Gelucire® 48/16 on the other hand, is a water-soluble surfactant with an onset melting temperature of 45 °C (PharmaExcipients, 2020b). Thus, once mixed with water, it resulted in a solid consistency that is well-suited for SSE 3D printing. Due to that, Gelucire® 48/16 was selected as the main matrix in this study.

Both water content and temperature are critical processing parameters for 3D printing with active antibodies and must be optimised (Mandon et al., 2016). On this basis, two formulations were prepared and studied (Table 1); the first formulation contained a mixture of water and coconut oil as plasticisers, wherein the amount of water was selected close to the minimum amount needed to obtain the required infliximab dose. In the second formulation, water was used alone as a plasticising agent. Different preparation temperatures were explored, wherein 60 °C was found as the optimum preparation temperature since it is the minimum temperature that results in the proper melting and mixing of all components. Both formulations, were successfully printed having well-defined structures and with consistencies suitable for handling (Fig. 1). Compared to the suppositories previously reported by Seoane-Viaño et al. (Seoane-Viaño et al., 2021a), the current suppositories were printed at much lower temperatures, indicating the suitability of purified water as a plasticising agent for SSE printing ink.

**Fig. 2:**
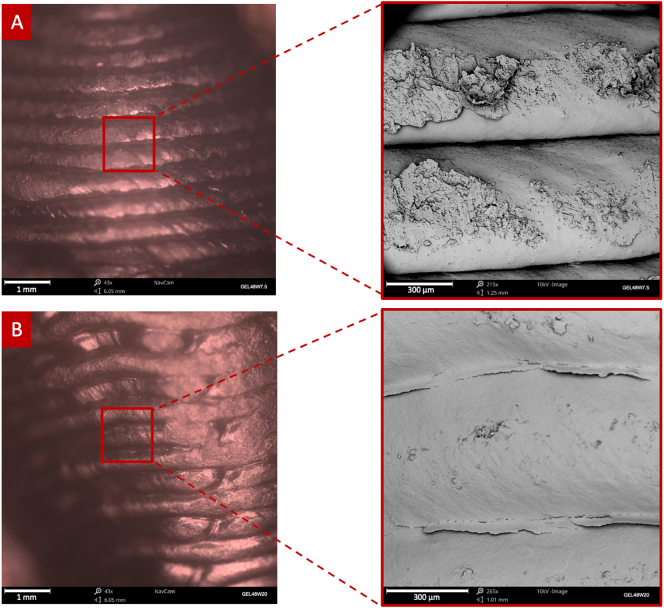
(left) Optical navigation camera and (right) SEM images of the surfaces of the (A) GEL48W7.5 and (B) GEL48W20 suppositories.

Optical and SEM images of the surface of the suppositories further verified the high printing resolution and strong structural integrity of the suppositories, indicated by the well-defined lines observed (Fig. 2). Compared to the GEL48W7.5 suppositories, the GEL48W20 ones showed smoother surfaces, which can be attributed to the higher water content in the formulation, resulting in the better mixing and dissolution of the content.

Both formulations resulted in suppositories with a mean weight of 2.14 g, with the GEL48W20 formulation resulting in less weight variation (Table 2). Similarly, both formulations resulted in suppositories with a mean layer height of 0.052 mm, which was higher than the theoretical layer height (0.5 mm). The mean drug loadings within the suppositories were 104.25 ± 1.67% and 104.80 ± 0.70% of the theoretical drug loading for the GEL48W7.5 and GEL48W20 suppositories, respectively. These values indicate that infliximab remained stable during the preparation and printing processes. The slightly higher drug loading may be attributed to water evaporation during the preparation and/or printing steps. In terms of binding capacity, it was found that 84.49 ± 4.54% and 64.84 ± 7.74% of the theoretical infliximab from the GEL48W7.5 and GEL48W20 suppositories can bind, respectively. The binding capacity of the GEL48W7.5 formulation was in fact higher than that of mAB-loaded 3D printed devices previously reported (∼69% binding capacity) (Carlier et al., 2021). Thus, indicating that the SSE technology may be more suitable for mABs compared to the fused deposition modelling (FDM) technology. No significant differences (*P* > 0.05) were established between the two formulations in statistical analysis.

**Table 2:** Results of the characterisation of the 3D printed suppositories, including weight, drug loading, binding capacity and disintegration time (mean ± SD).

Formulation	Weight (g)	Actual layer height (mm)	Theoretical drug dose (mg)	Actual drug dose (mg)	Drug loading (%)	Biding capacity (%)	Disintegration time (min)
GEL48W7.5	2.14 ± 0.04	0.52 ± 0.01	5.14 ± 0.06	5.36 ± 0.03	0.250 ± 0.004	84.49 ± 4.54	14.14 ± 0.09
GEL48W20	2.14 ± 0.01	0.52 ± 0.02	5.14 ± 0.01	5.39 ± 0.02	0.252 ± 0.002	64.84 ± 7.74	11.76 ± 0.36

**Fig. 3:**
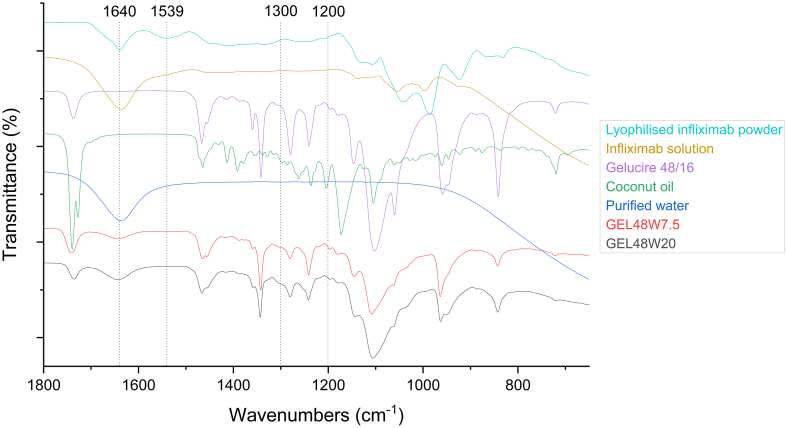
FTIR spectra of the lyophilised infliximab powder, reconstituted infliximab solution, the lipid excipients (Gelucire® 48/16 and coconut oil), purified water and 3D printed suppositories (GEL48W7.5 and GEL48W20).

FTIR spectroscopy was conducted to investigate infliximab's secondary structural changes (e.g., α-helix, β-sheet, β-turns or random coils) (Barth and Zscherp, 2002). Typically, mABs are characterised using their Amide spectral bands (Güler et al., 2016). This includes Amide I bands, which appear between 1600 and 1700 cm^−1^ (i.e., C=O stretching vibration and C—N groups), and Amide II bands, which appear between 1510 and 1580 cm^−1^ (i.e., N—H bending, C—N stretching vibration and C—C stretching vibration). According to previous studies, both Amide I and II bands are expected to be observed even at low concentrations of a biopharmaceutical (Corujo et al., 2018; Tiernan et al., 2020). As the Amide I region is mainly used for secondary structure analysis, β-sheet structures were identified when bands located at 1640 cm^−1^ and between 1690 to 1695 cm^−1^ (Tian et al., 2007). For α-helix and β-turns, bands between 1660 to 1650 cm^−1^ and 1690 to 1665 cm^−1^ were distinguished, respectively. FT-IR spectra of infliximab (both powder and solution) had characteristic vibrational bands at 1640 cm^−1^ (Amide I region) (Fig. 3), which correspond to the β-sheet structures of infliximab (Costantino et al., 1997; Faghihi et al., 2019). The same band was observed in the spectra of both 3D printed suppositories. However, this band is also characteristic of water, wherein the H–O–H bending vibrations at 1635 cm^−1^ overlap with the Amide I bands of the infliximab (Arrondo and Goñi, 1999). Amide II bands at 1539 cm^−1^ were observed in the infliximab powder, but they were less clear in the solution and absent in the spectra of 3D printed suppositories. Whilst it has been previously proposed that Amide III bands (i.e., between 1300 and 1200 cm^−1^) can be used to distinguish a mAB's secondary structure (Bal Ram et al., 1993), this region overlapped with the characteristic bands of the lipid components. Thus, it was not possible to distinguish infliximab's patterns in the 3D printed formulations.

The disintegration time of the suppositories was determined according to the EP, wherein disintegration was reached when the suppository collapsed or broke into small pieces. It was observed that the GEL48W20 suppositories disintegrated significantly faster (*P* < 0.05) than the GEL48W7.5 suppositories (Table 2), which was attributed to the much higher water content in the GEL48W20 formulation.

**Fig. 4:**
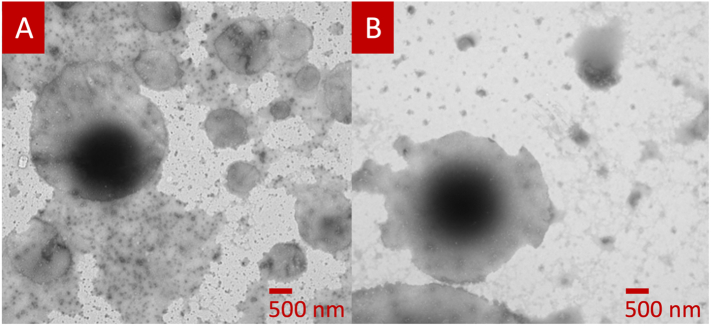
Representative TEM microphotographs of lipid droplets formed following the emulsification of the (A) GEL48W7.5 and (B) GEL48W20 suppositories, fixed with phosphotungstic acid. The black spots are traces of the phosphotungstic stain.

The self-emulsifying drug delivery systems (SEDDS; i.e., isotropic blends of oils, surfactants, co-surfactants and an API, which can create oil-in-water (O/W) emulsions that are kinetically stable) formed by the SSE suppositories were investigated and characterised using the self-emulsification time, globule size, ζ-Potential and PDI studies. The GEL48W7.5 formulation exhibited a slower self-emulsification time of 2.07 min compared to the GEL48W20 formulation that self-emulsified within 1.90 min (Table 3). No significant differences (*P* > 0.05) were determined from the statistical analysis of the self-emulsification time. The emulsions formed by the GEL48W7.5 suppositories were significantly smaller (*P* < 0.05) than those resulting from the GEL48W20 suppositories, with both having high PDIs that are indicative of heterogeneity (Deli et al., 2009). Interestingly, the average droplet size of the GEL48W7.5 emulsions (282.90 ± 9.58 nm) is quite close to the particle size identified by Mohan *et al.* as having the highest transport across inflamed caco-2 epithelial cell barriers (291.97 ± 10.92 nm) (Mohan et al., 2023). This suggests that transport of these emulsions can occur transcellularly, especially that inflammatory cytokines (e.g., IFN-γ and TNF) result in the re-arrangement of actin into stress fibres and promote endocytosis of tight junction proteins in IBD (Capaldo and Nusrat, 2009; Stolpen et al., 1986). ζ potential measures the surface potential and electrostatic repulsion between neighbouring particles in a dispersed system, and is used as indication for analysing the stability of emulsions. Typically, a ζ potential higher than 30 mV is indicative of a moderately stable emulsion, irrespective of the charge. This is because the presence of ample repulsive forces restricts aggregation and flocculation within a dispersion (Joseph and Singhvi, 2019). Accordingly, the GEL48W7.5 emulsions were found to be significantly more stable (*P* < 0.05), marked by their higher negative charge. Both emulsions were much smaller and more stable than those previously described by Seoane-Viaño et al. (Seoane-Viaño et al., 2021a). The morphology and structure of the formed droplets were assessed by capturing TEM microphotographs. The droplets are spherical in shape, with size ranges consistent with the data obtained from dynamic light scattering readings (Fig. 4).

**Table 3:** Characteristics of the of the emulsions formed by the 3D printed suppositories (mean ± SD).

Formulation	Self-emulsification time (min)	Droplet Size (nm)⁎	PDI⁎	ζ Potential (mV)⁎
GEL48W7.5	2.07 ± 0.42	282.90 ± 9.58	0.369 ± 0.03	−30.90 ± 0.87
GEL48W20	1.90 ± 0.56	429.8 ± 26.24	0.592 ± 0.04	−24.03 ± 0.51

⁎ *Statistically significant difference (P < 0.05).*

**Fig. 5:**
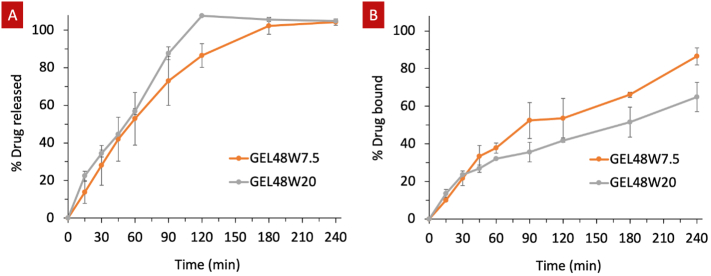
(A) *In vitro* drug release profiles obtained using SEC, and (B) binding capacity of infliximab following ELISA analysis, in 0.1 M phosphate buffer (pH 8.0).

Infliximab's dissolution rate from the suppositories was assessed via SEC and its biologic activity was evaluated by measuring its binding capacity using ELISA. SEC showed that 42.04 ± 11.71% and 44.58 ± 0.88% infliximab was released within 45 min from the GEL48W7.5 and GEL48W20 suppositories, respectively (Fig. 5). After 60 min, the GEL48W20 suppositories exhibited a more rapid drug release rate compared to the GEL48W7.5 suppositories. On the other hand, ELISA assay demonstrated that lower fractions of infliximab provide a biologic activity and actually remain active compared to the total infliximab released. In particular, the incorporation of water on its own in the GEL48W20 suppositories results in ∼65% binding, whereas the combination of water and oil in the GEL48W7.5 suppositories preserves ∼85% of infliximab's biologic activity. Herein, the loss of biologic activity implies that the remaining infliximab in the suppositories may have partially degraded during the preparation or printing processes. No significant statistical differences (*P* > 0.05) were observed between the two formulations' dissolution and binding characteristics.

To date, we have shown that SSE 3D printed suppositories can be exploited as a flexible drug delivery platform for a range of different molecules. Initially, we developed this platform to deliver small molecules, such as tacrolimus (Seoane-Viaño et al., 2020; Seoane-Viaño et al., 2021a), budesonide and tofacitinib citrate (Awad et al., 2023b), where the effect of changing the lipid component or drugs' content on the release properties was studied. On this basis, the current formulation was adapted and developed to allow the incorporation of larger drug molecules including biopharmaceuticals. It was demonstrated that with a small modification to the original formulation involving the addition of water, the mAB can be stabilised to allow it to withstand the printing and extrusion processes.

Previous studies have shown that patients prefer suppositories over enemas (Campieri et al., 1988) and foams (Farup et al., 1995). Furthermore, in a study comparing a budesonide foam versus a budesonide suppository, it was shown that the suppositories were equally efficacious in instigating mucosal healing in patients suffering from mild-to-moderate active ulcerative proctitis (i.e., idiopathic mucosal inflammation of the rectum) and provided superior clinical activity to the foam formulation (Kruis et al., 2022). In fact, almost half of the patients (46.9%) preferred the suppositories over the foams (22.1% preferred the foam and 28.5% had no preference), with the majority of patients stating that it was “easy” (76.9%) to administer the rectal formulations in the evening and that their daily routines were “almost not” (62.2%) affected by application in the morning. Thus, it can be deduced that the use of suppositories can have major advantages over other types of rectal formulations, especially in the case of distal (also known as left-sided) colitis.

Infliximab has been previously shown to be stable under colonic conditions (Yadav et al., 2016), which provided a basis for the current study. While there is no definite evidence that infliximab exerts a local effect in the rectum, a pilot study has shown that that infliximab can result in endoscopic and clinical improvement of patients' conditions when directly injected into the colorectal stricture of IBD patients who previously did not respond to infliximab infusions (Swaminath and Lichtiger, 2007). In another example, when an infliximab enema was compared to an IV injection in a dextran sodium sulphate (DSS) colitis mice model, it was shown that the enema resulted in a comparable improvement of the colitis (Lopetuso et al., 2013). Thus, it can be inferred that the infliximab is likely to provide a clinical action when administered in the form of a rectal suppository.

In the current work, although SEC showed that no infliximab loss occurred during the printing process, infliximab's full binding capacity could not be preserved following the printing process. Hence, more work is needed to optimise the proposed approach in an aim to maximise and preserve infliximab's binding affinity. This could include further modification to the printing ink to allow printing at lower temperature or incorporating stabilising agents such as sugars or polyols (Mensink et al., 2017). Herein, the ratio of water-to-oil was essential for determining infliximab's biological activity. This is potentially due to the fact that aqueous mAB formulations are susceptible to oxidation, deamination, aggregation and fragmentation (Daugherty and Mrsny, 2006), resulting in their reduced stability and affinity. This effect can therefore be counteracted through the incorporation of hydrophobic systems that help moderate water's effect (Marquette et al., 2014). As such, this explains why the addition of oil, which is hydrophobic in nature (Chandler, 2002), helps preserve infliximab's potency.

Whilst the loss of 15% of infliximab's bioactivity is a considerable amount, this is still considered an improvement over the 30% loss in activity previously reported by Carlier *et al.* in their study involving the fabrication of 3D printed implants using FDM 3D printing (Carlier et al., 2021). It should be noted however, that in this case it is not possible to directly compare both studies, as the mAB employed by Carlier *et al.* was not disclosed. Nevertheless, since the mAB employed by Carlier *et al.* has a MW of 150 kDa and infliximab's MW is 149 kDa (Hong et al., 2017; Knight et al., 1993), it can be anticipated that both mABs might behave similarly when subjected to the 3D printing process. Furthermore, the current findings have also shown more promising results compared to oral infliximab formulations previously reported, such as nanoparticles (i.e., 53.95 ± 19% and 80.60 ± 21% for the 292 and 594 nm particles, respectively (Mohan et al., 2023)) and nano-in-microparticles (i.e., 65.7 ± 3.5% bioactivity (Li et al., 2021)). An additional advantage to note is that because infliximab's bioactivity has been previously shown to decline with increased storage time (Maurer et al., 2016), the use of 3D printing could serve as a viable way for producing solid dosage forms containing infliximab at the point-of-care, avoiding the loss or reduction in its binding capacity and preserving its bioactivity.

## 4 Conclusions

This study demonstrates that SSE 3D printing can serve as a viable method for directly fabricating rectal dosage forms containing biologics. The suppositories were successfully printed with varying compositions, wherein the presence of infliximab in the suppositories was verified following the printing process. Depending on their water and oil content, the formulations displayed different emulsification properties and drug bioactivity. It was demonstrated that infliximab is released slightly faster from the GEL48W20 suppositories in comparison to the GEL48W7.5 suppositories. However, the emulsions formed by the GEL48W7.5 suppositories were more stable, with infliximab having a better binding capacity compared to when present in the emulsions formed by the GEL48W20 suppositories. Overall, this work shows that the rectal route has the potential to be used as an alternative way for delivering mABs, wherein patients could self-administer their own medications without the need for trained healthcare professionals. Additionally, it demonstrates the potential of 3D printing to be used as a novel manufacturing platform for biologics, setting the scene for new opportunities in the future. Nonetheless, further investigation is needed to amplify the technology's prospects.

## Funding

This project has received funding from the Interreg 2 Seas programme 2014-2020 co-funded by the 10.13039/501100008530European Regional Development Fund under subsidy contract “Site Drug 2S07-033”.

## Declaration of Competing Interest

Abdul W. Basit and Alvaro Goyanes are founders of the pharmaceutical company FabRx.

## Data Availability

Data will be made available on request.
